# Operational Characterization of Pressure Regulating Valves

**DOI:** 10.1155/2018/1213638

**Published:** 2018-06-06

**Authors:** M. V. Talamini Junior, A. C. S. de Araujo, A. P. de Camargo, E. Saretta, J. A. Frizzone

**Affiliations:** ^1^Biosystems Engineering Department, Luiz de Queiroz College of Agriculture (ESALQ), University of São Paulo (USP), Piracicaba, SP, Brazil; ^2^College of Agricultural Engineering (FEAGRI), State University of Campinas (UNICAMP), Campinas, SP, Brazil; ^3^UFSM, Cachoeira do Sul Campus, Cachoeira do Sul, RS, Brazil

## Abstract

The functionality of pressure regulating valves (PRVs) is important for proper uniformity and efficiency of irrigation during center pivot irrigation, especially when the center pivot operates on sloping terrain. In practice, the regulated pressure at the PRV outlet is slightly influenced by its inlet pressure, the flow rate through it, and hysteresis effects. The objectives of this work were (a) to evaluate operational characteristics of PRVs based on requirements stated by ISO 10522 (1993) and (b) to model the regulated pressure as a function of inlet pressure and flow rate through the valve considering hysteresis. We carried out tests to evaluate regulation uniformity, regulation curve, hysteresis, and the regulated pressure as function of flow rate and inlet pressure. The following three models of PRVs were evaluated: 10 PSI, 15 PSI, and 20 PSI. For each model, three samples were tested under increasing and decreasing conditions of inlet pressure, within the range from 49.03 to 784.53 kPa, with increments of 49.03 kPa. In addition, flow rates were tested within the range of 0 and 4 m^3^ h^−1^. From the gathered data, models to predict outlet pressure as a function of inlet pressure and flow rate were fitted.

## 1. Introduction

The center pivot irrigation system has advantages when compared to other systems, especially for use in large areas [1]. With significant advantages of time and labor saving and possibility of chemigation and precision irrigation, the center pivot system has been used in some high labor costs countries as well as in developing countries [2]. This irrigation equipment is designed to apply water uniformly along its axis, though factors such as topography, ambient temperature, and wind speed may influence irrigation performance. The application uniformity must remain satisfactory to ensure efficient use of resources like water and energy, lower their costs, and properly supply the crop water requirements [3].

Pressure varies along the lateral line of center pivots mainly because of pressure losses, that is, friction and minor losses, and topography. Center pivots generally use pressure regulating valves (PRVs) installed at the inlet of each emitter to ensure steady pressure and constant discharge [4]. Such a technique, although it involves some waste of energy owing to pressure dissipated by the regulating valves, is quite useful to attain satisfactory levels of application uniformity. The most common models are spring valves, in which a frame enfolds a piston [5, 6]. In most of cases, only center pivots installed on flat terrain may be designed without PRVs.

Pressure regulators do not store or produce energy; hence, the outlet pressure cannot exceed the inlet pressure [7]. Although PRVs are designed to maintain constant outlet pressure regardless of the inlet pressure and flow rate, in practice, both variables may influence the outlet pressure, that is, the regulated pressure. If the pressure regulating valves do not operate properly, the application uniformity may be unsatisfactory and there will be waste of energy due to pressure losses caused by the valves. ISO 10522 [8] is a standard that states testing methods, requirements, and criteria that must be met by pressure regulating valves used in irrigation. The evaluation of irrigation equipment following standardized methods is important to determine operational characteristics of the equipment and to quantify its performance.

The objectives of this work were as follows: (a) to evaluate operational characteristics of PRVs based on requirements stated by ISO 10522 (1993) and (b) to model the regulated pressure as a function of inlet pressure and flow rate through the valve considering hysteresis. We carried out tests to evaluate regulation uniformity, regulation curve, and the regulated pressure as function of flow rate and inlet pressure. Tests were performed in a laboratory according to the standard and recommendations of the PRV manufacturer. This work extends a previous research accomplished by [9].

## 2. Materials and Methods

### 2.1. Testing Bench

**Figure 1:**
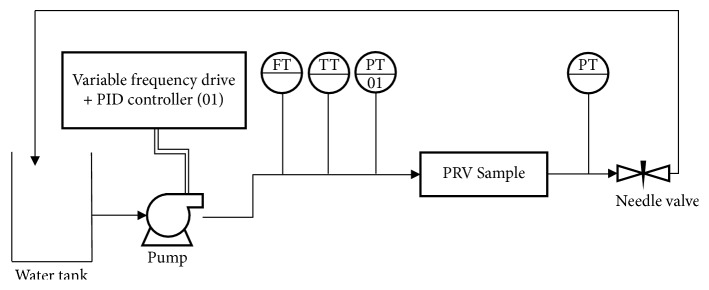
Instrumentation flowchart of the testing bench for evaluation of pressure regulating valves (FT: flow transmitter; TT: temperature transmitter; PT: pressure transmitter) [9].

Tests were undertaken in a testing bench (Figure 1) consisting of a pump and an automated system for pressure control, equipped with a variable frequency drive and proportional-integral-derivative (PID) controller. The PID controller uses a pressure transmitter, installed at the inlet of the PRV, to calculate the error between the pressure set point and measured pressure and then applies an output signal to the variable frequency drive. The PRV to be tested is installed downstream of the electromagnetic flowmeter (0 to 4 m^3^ h^−1^, expanded uncertainty of 0.5%). The pressures at the inlet and outlet of the PRV were monitored using pressure transmitters (inlet pressure: 0 to 980.66 kPa, expanded uncertainty of 0.10%; outlet pressure: 0 to 490.33 kPa, expanded uncertainty of 0.10%). The water temperature was monitored using a temperature transmitter (0 to 50°C, expanded uncertainty of 0.5°C). A needle valve was installed downstream of the PRV to enable manual setting of the flow rate through the PRV.

### 2.2. Material Evaluated

Three models of pressure regulating valves were evaluated: (a) 10 PSI, (b) 15 PSI, and (c) 20 PSI. The models are named according to their declared preset pressure. The declared preset pressure corresponds to the pressure at the outlet of the pressure regulator operated under a reference flow velocity of 1 m s^−1^ [8]. According to the manufacturer, the Brazilian company Fabrimar, the three models present a nominal pressure of 784.53 kPa. The nominal pressure corresponds to the maximum static working pressure at which the PRV is stated to operate under normal service conditions [8]. In addition, all models have 3/4′′ female threaded connections at the inlet and outlet of the valve.

### 2.3. Regulation Uniformity

Twenty units of each model of PRV were evaluated in this test. The regulated pressure (i.e., the pressure at the PRV outlet) was measured under an inlet pressure 1.5 times the declared preset pressure and at a flow rate corresponding to a reference velocity of 1 m s^−1^, as recommended by ISO 10522. Based on the declared preset pressures, tests were carried out under the following inlet pressures: (a) model 10 PSI = 102.97 kPa; model 15 PSI = 154.94 kPa; and model 20 PSI = 206.92 kPa.

From the obtained results, the coefficient of variation (*CV*) was calculated according to the following equation:

(1) $$\begin{array}{c} CV\left(\;\%\;\right)=100\frac{S_{p}}{\overset{-}{p}} \end{array}$$

where *S*_*p*_ is the sample standard deviation of the regulated pressures; $\overset{-}{p}$ is the mean regulated pressure of the sample.

As the PRV models evaluated were classified as ordinary pressure regulators, the mean regulated pressure ($\overset{-}{p}$) shall not deviate from the declared preset pressure by more than 7%, and the coefficient of variation shall not be greater than 10% [8].

### 2.4. Regulation Curve

Three units of each model of PRV were evaluated in this test. The regulated pressure was measured at constant inlet pressure and under flow rates corresponding to the following reference velocities: 0.0, 0.5, 1.0, 1.5, and 2.0 m s^−1^ [8]. The manufacturer requested the laboratory to submit the PRVs to a flow rate of 3 m^3^ h^−1^. Thus, the flow rates tested were 0.00, 0.57, 1.13, 1.70, 2.26, and 3.00 m^3^ h^−1^.

For each model of PRV, a chart of regulated pressure as a function of flow rate was plotted. The chart comprises three series of data corresponding to tests carried out under the following inlet pressures: (a) 1.5 times the declared preset pressure; (b) 0.8 times the nominal pressure; and (c) the inlet pressure at the middle of the regulation range [8]. Thus, 10 PSI model was evaluated under 102.97, 627.63, and 441.30 kPa; the 15 PSI model was evaluated under 154.94, 627.63, and 416.78 kPa; and the 20 PSI model was evaluated under 206.92, 627.63, and 441.30 kPa.

By increasing the reference velocity by 1 m s^−1^, from 0.5 to 1.5 m s^−1^, and from 1 to 2 m s^−1^, ISO 10522 [8] states that the regulated pressure shall not vary by more than 10% from the declared preset pressure for PRVs of accuracy level A and 20% for PRVs of accuracy level B.

### 2.5. Regulated Pressure as Function of Flow Rate and Inlet Pressure

Three units of each model of PRV were evaluated in this test. ISO 10522 [8] describes a test to determine the regulated pressure as function of inlet pressures at constant flow rate and neglecting hysteresis effects. The manufacturer claims that the regulated pressure of PRVs is influenced by the flow rate through the valve, and the recommended procedure by ISO does not factor in such an effect. Moreover, hysteresis effects are expected to influence the regulated pressure and should be taken into account while modeling the regulated pressure as a function of inlet pressure and flow rate.

Inlet pressures ranged from 49.03 to 784.53 kPa in intervals of 49.03 kPa. Tests were carried out systematically increasing and decreasing inlet pressures in order to factor in hysteresis effects. Owing to the manufacturer specifications, the 10 PSI model was evaluated under flow rates of 0.57, 1.13, 1.70, 2.26, 3.00, and 3.60 m^3^ h^−1^. Similarly, for the 15 PSI and 20 PSI models, the same values were tested, except the last one, which was 4.00 m^3^ h^−1^.

Experimental data gathered during the tests were employed to fit a mathematical model for each PRV model. The mathematical model enables estimation of the regulated pressure as a function of flow rate and inlet pressure (see (2)) [4].

(2) $$\begin{array}{c} P=a+bQ+\frac{c}{1+e^{\left[\left(d-\left(P_{in}/98.066\right)\right)/f\right]}} \end{array}$$

where *P* is the regulated pressure (kPa); *P*_*in*_ is the inlet pressure (kPa); and *Q* is the flow rate through the PRV (m^3^ h^−1^); *a*, *b*, *c*, *d*, *f* are coefficients. The constant 98.066 was used for converting pressure units from kgf cm^−2^ to kPa.

The coefficients of (2) were determined using the least-square method and the solver supplement of Microsoft Excel®. The goodness of fit was assessed based on the Root Mean Square Error (RMSE), 1:1 straight line, and distribution of frequency of errors. RMSE was calculated as follows:

(3) $$\begin{array}{c} RMSE=\sqrt{\frac{\sum_{i=1}^{N}{\left(\hat{P}-P\right)}^{2}}{N}} \end{array}$$

where $\hat{P}$ is the estimated value of regulated pressure; *P* is the measured regulated pressure; and *N* is the number of pairs of values.

The relative error between estimated and measured values of pressure (*δ*) was determined by the following equation:

(4) $$\begin{array}{c} \delta \left(\;\%\;\right)=100\frac{\left|\hat{P}-P\right|}{P} \end{array}$$

## 3. Results and Discussion

### 3.1. Regulation Uniformity

Table 1 shows results from the regulation uniformity test. The coefficient of variation was smaller than 10% for all the evaluated models, and thus the PRVs meet one of the criteria stated by ISO 10522. The low values of CV indicate a proper quality control on the manufacturing process. The regulated pressure was similar when twenty samples of each model were evaluated under the same testing conditions.

The deviation between the average regulated pressure and the declared preset pressure was lower than 7% for the 15 PSI and 20 PSI models and higher than that threshold for the 10 PSI model. Therefore, only the 10 PSI model did not meet one of the criteria defined by ISO 10522. For this PRV model to comply with the standard, the declared preset pressure could be changed to a value closer to 60.8 kPa (8.8 PSI).

### 3.2. Regulation Curve

**Figure 2:**
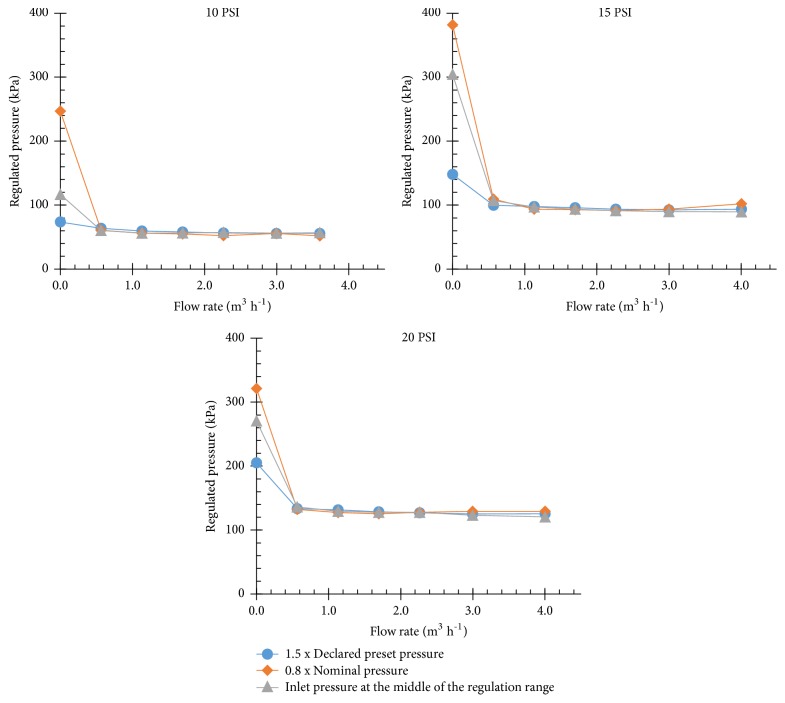
Regulation curves.

Figure 2 shows the regulation curves obtained from testing the three PRV models. The regulated pressures at flow rates higher than zero were similar under the three inlet pressures evaluated. This indicates that all models operated properly under a range of testing conditions. The regulated pressure at zero flow was slightly higher than the declared preset pressure, but it is tolerated by the standard.

**Figure 3:**
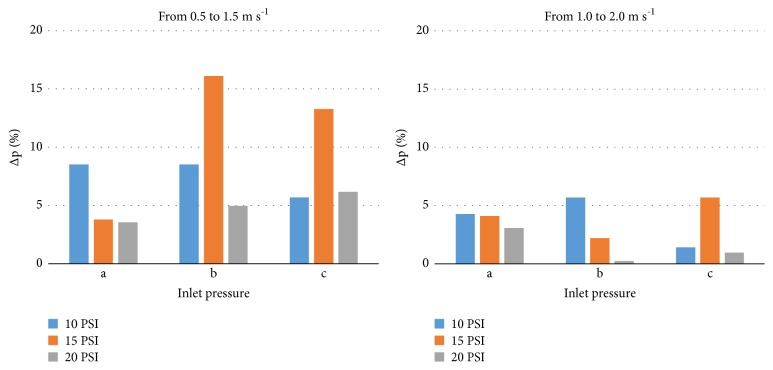
Deviation between the regulated pressure and the declared preset pressure (Δp) when the reference flow velocity varied from 0.5 to 1.5 m s^−1^ and from 1.0 to 2.0 m s^−1^. Inlet pressures: (a) 1.5 times declared preset pressure; (b) 0.8 times nominal pressure; and (c) inlet pressure at the middle of the regulation pressure range.

Figure 3 shows the deviation between regulated pressure and declared preset pressure when the reference flow velocity varied from 0.5 to 1.5 m s^−1^ and from 1.0 to 2.0 m s^−1^. Regarding the “regulation curve” requirement, the results shown in this figure enable classification of the models according to accuracy level [8]. The deviation of values for the 10 PSI and 20 PSI models was always lower than 10%; hence, these models may be classified as pressure regulators with an accuracy level A [8]. However, the 15 PSI model presented values lower than 20%, and thus it may be considered a pressure regulator with an accuracy level B [8]. The same results were reported by Lima et al. [4] for another 20 PSI model, similar to the one tested. The authors found an accuracy level A when operating within the flow range of 1.15 to 3.40 m^3^ h^−1^ and level B when the flow rate was outside this interval.

### 3.3. Hysteresis

**Figure 4:**
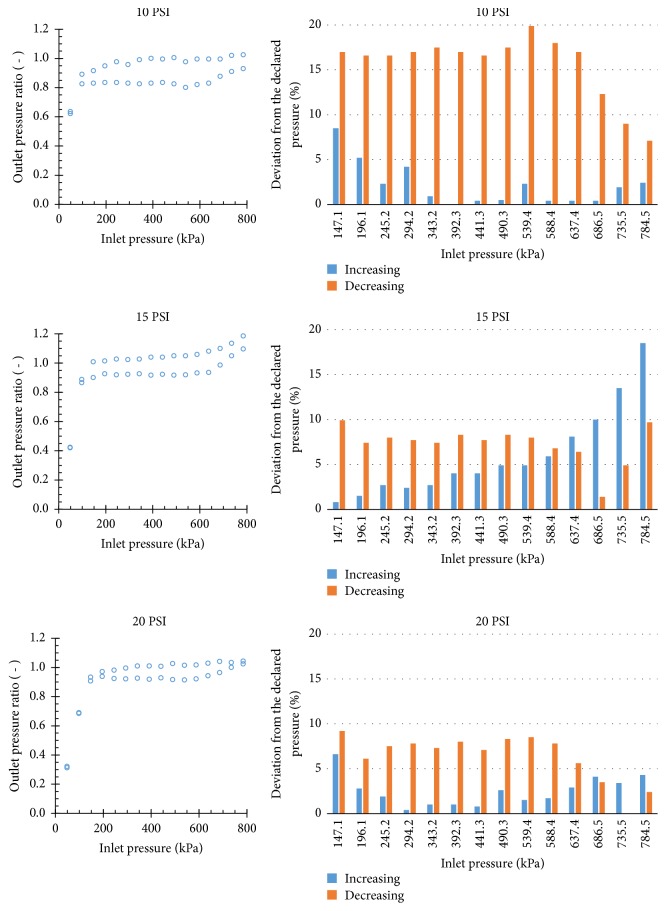
Hysteresis observed when operating the PRVs under flow rate of 1.13 m^3^ h^−1^ (flow velocity of 1 m s^−1^).

Figure 4 shows results obtained by increasing and decreasing the inlet pressure under a constant flow rate of 1.13 m^3^ h^−1^, which corresponds to the reference flow velocity of 1 m s^−1^. As expected, the outlet pressure did not track the same line on decreasing pressure as it did on increasing pressure owing to internal friction, and such behavior is called hysteresis [7]. According to ISO 10522, hysteresis of PRVs must be determined under a single reference flow velocity of 1 m s^−1^. In addition, results obtained from those tests must be used for classifying the accuracy level of PRVs.

The relative deviations between the declared preset pressure and the regulated pressure under increasing and decreasing inlet pressures are also shown in Figure 4. Maximum deviations from the declared preset pressure reached 19.9%, 18.5%, and 9.2%, respectively, for the 10 PSI, 15 PSI, and 20 PSI models. Based on the results, the 10 PSI and 15 PSI models presented values lower than 20%, and thus they may be considered as pressure regulators of accuracy level B [8]. However, the 20 PSI model presented deviation from the declared preset pressure lower than 10%; hence, it may be classified as a pressure regulator of accuracy level A [8].

**Figure 5:**
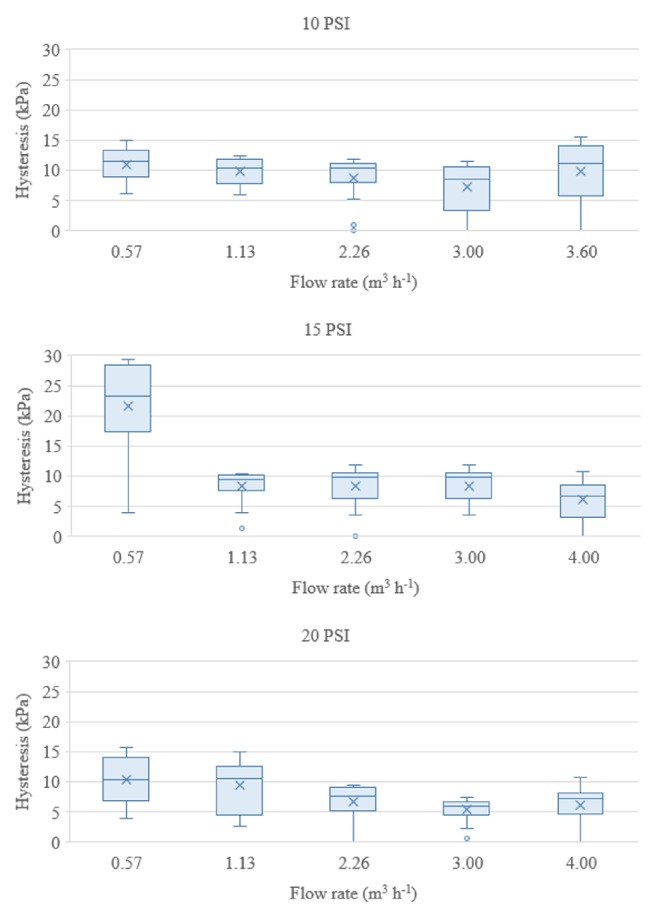
Boxplots presenting values of hysteresis for each model of PRV over the five flow rates evaluated: (a) 0.57 m^3^ h^−1^; (b) 1.13 m^3^ h^−1^; (c) 2.26 m^3^ h^−1^; (d) 3.00 m^3^ h^−1^; (e) 3.60 m^3^ h^−1^ (10 PSI) or 4.00 m^3^ h^−1^ (15 PSI and 20 PSI).

In addition to ISO 10522 requirements, we also evaluated how hysteresis varied under lower and higher flow velocities.  Figure 5 shows boxplots indicating the deviation between values of regulated pressure obtained under increasing and decreasing inlet pressures, that is, hysteresis, considering five flow rates. The maximum hysteresis was 15.6, 29.4, and 15.7 kPa for the 10 PSI, 15 PSI, and 20 PSI models, respectively. Such values of hysteresis divided by the corresponding declared preset pressure of each PRV model result in the following relative values that represent relative deviation from the declared preset pressure: 22.6% (10 PSI), 28.4% (15 PSI), and 11.4% (20 PSI). The higher hysteresis observed when evaluating the 15 PSI model might be related to higher friction between internal parts of the valve [7]. Supposing a PRV is installed upstream of an emitter of turbulent flow, which typically has an exponent of flow about 0.5 [10], deviations in the discharge of the emitter due to hysteresis would reach the following maximum values: 10.7% (10 PSI), 13.3% (15 PSI), and 5.5% (PSI). However, perhaps analyzing the average values of hysteresis for each model of PRV could be more realistic than the maximum values. The average values of hysteresis were 9.3, 11.1, and 7.6 kPa within the full range of flow rates evaluated. Such values of hysteresis divided by the declared preset pressure of each PRV model result in 13.4% (10 PSI), 10.7% (15 PSI), and 5.5% (20 PSI); and the corresponding impact of hysteresis on the discharge of a turbulent flow emitter would be 6.5% (10 PSI), 5.2% (15 PSI), and 2.7% (20 PSI). Undoubtedly, such imperfections on the regulated pressure affect the emitter's discharge, diminishing efficiency and uniformity of irrigation, though quantifying such effects could be difficult.

### 3.4. Regulated Pressure as Function of Flow Rate and Inlet Pressure: Experimental Data

The regulated pressure of a PRV cannot exceed its inlet pressure, because the device does not produce nor store energy [7]. Ideally, a PRV is designed to maintain a constant regulated pressure regardless of the inlet pressure and flow rate. In practice, PRVs have friction between internal parts and constructive imperfections that prevent ideal operational characteristics. The regulated pressure is mainly influenced by the inlet pressure, flow rate, and hysteresis. When the inlet pressure is lower than the declared preset pressure, the outlet pressure must be lower than the inlet pressure due to friction.

**Figure 6:**
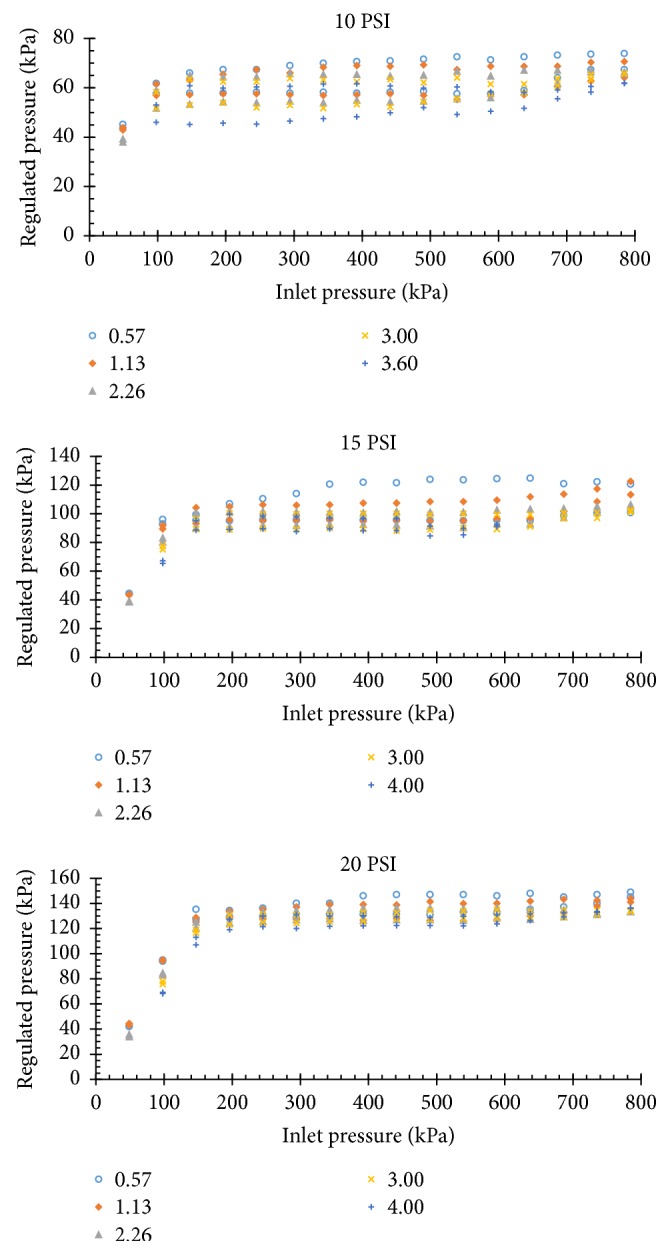
Data collected to fit mathematical models of regulated pressure as a function of flow rate and inlet pressure considering hysteresis.

Data gathered during the experiments and used for fitting coefficients of (2) are shown in Figure 6, which presents values of regulated pressure obtained when increasing and decreasing inlet pressures. In addition, Figure 6 presents a data series for each one of the flow rates evaluated. The regulated pressure is clearly influenced by the flow rate through the PRV. For all models of PRVs, higher flow rates resulted in lower regulated pressures because friction losses increase according to flow velocity. Furthermore, for a given flow rate, higher values of regulated pressure were observed when increasing inlet pressures as a result of hysteresis effects related to operational and constructive characteristics of the evaluated valves.

ISO 10522 [8] describes a test to determine the regulated pressure as a function of inlet pressure at constant flow rate. The manufacturer claimed taht the regulated pressure of PRVs was influenced by the flow rate through the valve, and the recommended procedure by ISO did not factor in such an effect. Based on the result shown in Figure 6, we consider that the next revision of ISO 10522 should include a test to determine the regulated pressure as a function of inlet pressure and flow rate considering hysteresis effects.

Proper performance of a center pivot equipped with PRVs requires that regulated pressure remains as steady as possible to ensure adequate uniformity and efficiency of irrigation. Within the operational range recommended by the manufacturer, the curve of regulated pressure as a function of inlet pressure for a constant flow rate should be as flat as possible. In addition, when comparing a series of curves for various flow rates, those curves also should be close to each other. Both characteristics are remarkable when designing large-size center pivots installed over nonuniform terrain. The slope of a center pivot lateral line changes as the equipment moves over the terrain, and it may cause changes in the pressure head at the inlet of the PRVs. The uniformity of application and the average amount of water applied by each sprayer may be influenced by the proper operation of the PRVs. Valves operating with regulated pressure near the declared preset pressure, regardless of other variables, may lead to proper operation of the center pivot, as the designed and the actual operational characteristics will match.

### 3.5. Regulated Pressure as Function of Flow Rate and Inlet Pressure: Modeling

Table 2 presents the coefficients of equations to estimate regulated pressure as a function of flow rate and inlet pressure, as well as the RMSE values and the limits of use for each model. Operating PRVs outside the limits described in Table 2 may lead to regulated pressures different from the declared preset values, which might cancel out the benefits expected from the PRVs.

**Figure 7:**
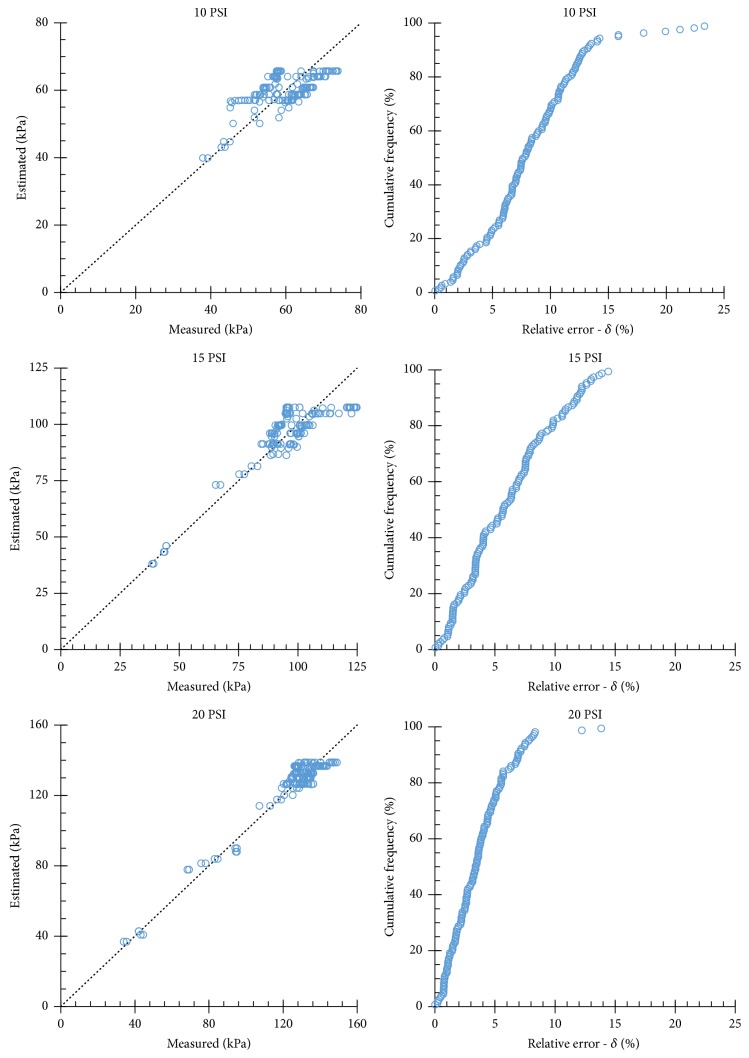
Measured and estimated values of regulated pressure and cumulative frequency of relative errors on estimating regulated pressure.

Figure 7 enables comparison between measured and estimated values of regulated pressure, enabling quantification of the accuracy of the proposed mathematical models. The charts with a straight 1:1 line enable comparison between estimated and measured values of regulated pressure. The charts presenting cumulative frequency of relative errors on estimating regulated pressure enable quantification of the accuracy of the proposed model. Analyzing the 10 PSI model, 95% of the estimated values presented relative errors lower than 15.9%, and 67.5% of estimated values presented relative errors of up to 10%. Analyzing the 15 PSI model, 95% of the estimated values presented relative errors lower than 12.6%, and 82.6% of the estimated values presented relative errors of up to 10%. Finally, analyzing the 20 PSI model, 95% of the estimated values presented relative errors lower than 7.9%; hence, this mathematical model presented the best accuracy among the three evaluated models.

Equation (2), which is used to estimate regulated pressure, follows a logistic function with a sigmoid curve. As the inlet pressure approaches zero, the regulated pressure also tends to zero. Through the initial stage of regulation, the outlet pressure is approximately exponential. Overcoming the inferior limit and entering the full regulation, the outlet pressure is not expected to change significantly with variations in inlet pressure. Thus, when the inlet pressure tends to the positive infinite, the regulated pressure becomes constant (i.e., *P* = *a* + *bQ* + *constant*). However, in real conditions, the PRV spring has limits of load, responding up to a superior limit of inlet pressure, above which the outlet pressure increases again. Therefore, the limits of regulation are necessary for adequate operation in the field. Zhang and Li [6] emphasized that the initial regulation pressure and preset pressure are important also in terms of performance of the PRV.

Vibration on the spring of the 15 PSI PRVs model was observed under flow rates of 4 m^3^ h^−1^ combined with inlet pressures higher than 686.46 kPa. One of the evaluated units from the 10 PSI model also presented such phenomena when operated at conditions of 3.6 m^3^ h^−1^ combined with inlet pressures higher than 686.46 kPa. None of the evaluated units from model 20 PSI presented such problems. When vibration of a spring occurs, a noise is produced by the valve, and the regulated pressure becomes highly unstable. As PRVs fail to regulate pressure when vibration occurs, they must not be operated under extreme conditions that may lead to spring vibration. Lima et al. [4] also reported vibration on the spring of a 20 PSI model of a PRV when the inlet pressure exceeded 588.40 kPa at flow rates higher than 3.4 m^3^ h^−1^.

## 4. Conclusion

The evaluated models of pressure regulating valves operated properly within the operating range recommended by the manufacturer and verified throughout indoors tests.

During the regulation uniformity tests, the coefficient of variation was smaller than 10%, complying with ISO 10522 requirements. The low values of CV indicate a proper quality control on the manufacturing process. In this test, the deviation between the average regulated pressure and the declared preset pressure was lower than 7% for the 15 PSI and 20 PSI models and higher than that threshold for the 10 PSI model. Although the 10 PSI model did not meet one of the criteria defined by ISO 10522, such nonconformity might be easily solved by changing the declared preset pressure to a value closer to 60.8 kPa (8.8 PSI). Another option might be redesigning the springs, though it would be more difficult for the manufacturer to do so.

During tests to determine regulation curves, all models presented regulated pressure slightly higher than the declared preset pressure at zero flow, but it is tolerated by the standard. Regarding ISO 10522 requirements related to regulation curves, the 10 PSI and 20 PSI models were classified as pressure regulators with accuracy level A, while the 15 PSI model was classified with level B.

During tests to quantify hysteresis of regulated pressure due to increasing and decreasing conditions of inlet pressure under constant flow rate, the 10 PSI and 15 PSI models were considered as pressure regulators with accuracy level B, while the 20 PSI model was classified with level A.

Combining results from regulation curves and hysteresis, only the 20 PSI model may be classified as a pressure regulator with accuracy level A; the other two models might be classified with level B.

The regulated pressure clearly was influenced by the inlet pressure and the flow rate through the PRV, as well as by hysteresis effects. Equations were fitted to predict regulated pressure as a function of inlet pressure and flow rate taking into account hysteresis. The model used to estimate regulated pressure seems to be appropriate. Finally, we consider that further revision of ISO 10522 should include a test to determine the regulated pressure as a function of inlet pressure and flow rate.

## Figures and Tables

**Table 1:** Results from the regulation uniformity test operated under the reference flow velocity of 1 m s^−1^.

Parameter	PRV model		
	10 PSI	15 PSI	20 PSI
Declared preset pressure (kPa)	68.65	102.97	138.27
Mean regulated pressure (kPa)	60.80	103.95	134.35
Standard deviation of the regulated pressures (kPa)	2.128	2.001	3.579
Coefficient of variation (%)	3.5	1.9	2.7
Deviation between the mean regulated pressure and the declared preset pressure (%)	11.5	0.7	2.3

**Table 2:** Coefficients of the equations used to estimate regulated pressure as a function of flow rate and inlet pressure.

Model	Equation coefficients					RMSE	Limits of use	
	*a*	*b*	*c*	*d*	*f*		*Q* (m^3^ h^−1^)	*P*_*in*_ (kPa)
10 PSI	−5.0871	−0.0292	5.7730	−0.9202	0.4357	0.0547	0.57 to 3.65	49.03 to 784.53
15 PSI	−3.5686	−0.0483	4.6925	−0.2054	0.3775	0.0739	0.57 to 4.00	49.03 to 784.53
20 PSI	0.2162	−0.0361	1.2187	0.8951	0.2819	0.0530	0.57 to 4.00	49.03 to 784.53
